# Bone mineral density and turnover response to GLP-1 receptor agonists in older adults with overweight/obesity and prediabetes/type 2 diabetes: a 20-week pilot trial *post hoc* analysis

**DOI:** 10.3389/fragi.2025.1691007

**Published:** 2025-11-27

**Authors:** Lauren Dinkla, Kristen M. Beavers, Ronna Robbins, Dela Akpalu, Sarah J. Wherry, Gary Miller, Daniel P. Beavers, Sara Espinoza, Jonathan Trejo, Allison Stepanenko, Tiffany M. Cortes

**Affiliations:** 1 Department of Health and Exercise Science, Wake Forest University, Winston-Salem, NC, United States; 2 Department of Nutrition and Food Sciences, Texas Woman’s University, Denton, TX, United States; 3 Department of Statistical Science, Wake Forest University, Winston-Salem, NC, United States; 4 Division of Geriatric Medicine, University of Colorado Anschutz Medical Campus, Aurora, CO, United States; 5 VA Eastern Colorado Geriatric Research, Education, and Clinical Center (GRECC), VA Eastern Colorado Healthcare System, Aurora, CO, United States; 6 Center for Translational Geroscience, Department of Medicine, Cedars-Sinai Medical Center, Los Angeles, CA, United States; 7 Diabetes and Aging Center, Department of Medicine, Cedars-Sinai Medical Center, Los Angeles, CA, United States; 8 Department of Medicine, UT Health San Antonio; Sam and Ann Barshop Institute for Longevity and Aging Studies, UT Health San Antonio, San Antonio, TX, United States; 9 Endocrinology Section, South Texas Veterans Healthcare System, San Antonio, TX, United States; 10 San Antonio Geriatric Research, Education, and Clinical Center (GRECC), South Texas Veterans Healthcare System, San Antonio, TX, United States

**Keywords:** glucagon‐like peptide‐1 receptor agonist, randomized controlled trial, older adults, bone mineral density, bone turnover

## Abstract

The purpose of this exploratory *post hoc* analysis was to study the impact of semaglutide on measures of bone health in older adults. Data were collected from a 20-week pilot trial (NCT05786521), which randomized 20 older adults (72.7 ± 4.8 years of age, 50% women, 45% Hispanic) living with prediabetes/diabetes (hemoglobin A1C 5.7%–7.5%) and overweight/obesity [body mass index (BMI): 32.9 ± 4.0 kg/m^2^] to 1.0 mg/weekly semaglutide + lifestyle counseling (n = 10) or lifestyle counseling alone (n = 10). The total body weight, bone mineral density (BMD), and bone turnover markers (BTMs) [C-terminal telopeptide of type 1 collagen (CTX) and procollagen type I N-propeptide (P1NP)] were measured at baseline and 20 weeks. Twenty-week weight loss was greater in the semaglutide + lifestyle counseling group than in lifestyle counseling alone (−5.3% vs. −0.89%; p < 0.01). No significant differences in whole-body BMD (p = 0.77) or BTMs (CTX: p = 0.56, P1NP: p = 0.78) were observed between groups over time. In this 20-week pilot trial, we did not find evidence to suggest that weight loss achieved with semaglutide was associated with change in BMD or BTMs in older adults. Notably, the observed differences showed consistently lower BMD and higher bone turnover at follow-up in the semaglutide + lifestyle group than in the lifestyle alone. Additional work in this area is warranted to further evaluate the effect of glucagon-like peptide-1 receptor agonist (GLP1Ra) use on skeletal health outcomes in older adults, given the pilot nature of the trial, the small degree of weight loss achieved, and the well-described association between weight loss and fracture risk.

## 1 Introduction

Obesity, defined as a body mass index (BMI) of 30 kg/m^2^ or greater, affects nearly 40% of older adults (e.g., aged 60+ years) in the United States (Emmerich et al., 2024). Excess adiposity is associated with an increased risk of several comorbidities across the lifespan, with older adults being particularly vulnerable to obesity-related functional decline (Kochanek, 2024). Although weight loss can improve chronic conditions such as hypertension, diabetes, and mobility disability, it presents a unique challenge for older adults because of its potential negative impact on bone health. Indeed, data from observational studies and lifestyle-based interventions consistently suggest that weight loss is associated with reduced bone mineral density (BMD) and increased fracture risk in older adults (Cortes et al., 2025), warranting attention and potential intervention (Villareal et al., 2005).

There have been significant advances in the management of obesity over the past several years. These include the development of next-generation glucagon-like peptide-1 receptor agonists (GLP1Ras), with their weight loss efficacy ranging from 12% to 15% for semaglutide and 20% for tirzepatide (Ghusn and Hurtado, 2024; Kim et al., 2021). Moreover, demonstrated health benefits of this medication class have grown beyond diabetes management to include additional Food and Drug Administration (FDA) indications for reduced cardiovascular and chronic kidney disease risk (Ozempic, 2024), along with obstructive sleep apnea (ZEPBOUND, 2024). Collectively, these shifts have led to an increasing number of older adults turning to GLP1Ras as a strategy for weight and disease management (Montero et al., 2024).

One salient concern regarding the use of GLP1Ras for older adults is their impact on skeletal health (Papageorgiou et al., 2019). So far, preclinical studies suggest that GLP1Ra-based therapies may enhance bone health by promoting bone formation and reducing bone turnover (Daniilopoulou et al., 2022; Tan et al., 2025). Clinical data on the effects of GLP1Ra use on human bone metabolism are largely neutral, showing no consistent effects on bone turnover markers (BTMs), bone mineral density, or fracture risk (Daniilopoulou et al., 2022), with emerging studies showing a potential for reduced fracture risk (Xie et al., 2025; Zhang et al., 2025).

Given the rapid and increasing uptake of GLP1Ra-based therapies, coupled with the knowledge gap surrounding their impact on clinically relevant bone outcomes in older adults, we conducted an exploratory *post hoc* analysis of a pilot trial to explore the impact of 20 weeks of semaglutide use on bone density and turnover in older adults with overweight or obesity.

## 2 Materials and methods

### 2.1 Study overview

The Effect of GLP1 Receptor Agonists on Physical Function, Body Composition, and Markers of Aging in Older Adults trial was conducted from April 2023 through May 2024 at the Sam and Ann Barshop Institute for Longevity and Aging Studies, University of Texas Health Science Center at San Antonio. Details of the design and methods are previously published (Cortes et al., 2024). In brief, the primary objectives were to evaluate the effects of semaglutide on the lean mass, physical function, and molecular biomarkers of aging in older adults with prediabetes or type 2 diabetes and overweight or obesity. The biomarkers of aging were analyzed in blood, adipose tissue, and skeletal muscle. The study is registered on clinicaltrials.gov (NCT05786521; PI: Cortes) and was approved by the Institutional Review Board at the University of Texas Health Science Center at San Antonio; all participants provided written informed consent.

### 2.2 Participants

Participants were recruited through the Barshop Call Center Registry, electronic medical records, community engagement activities, and web-based advertisements, and they underwent an in-clinic screening evaluation, detailed elsewhere (Cortes et al., 2024). In brief, individuals were eligible for inclusion if they were men or postmenopausal women, were aged 65 years or older, had a BMI of 27–40 kg/m^2^, had a hemoglobin A1C of 5.7%–7.5% or a fasting blood glucose greater than 100 mg/dL, were community-dwelling, and were willing and able to comply with the protocol requirements. Those with a history of cardiovascular events, uncontrolled hypertension, type 1 diabetes, ≥5% weight change 3 months prior to screening, impaired renal function (GFR≥29), or medication contraindications (personal or a family history of medullary thyroid cancer or multiple endocrine neoplasia syndrome type 2, history of pancreatitis, or diabetic retinopathy) were excluded.

### 2.3 Interventions

Eligible participants were randomized 1:1 into one of two groups (lifestyle counseling alone or semaglutide + lifestyle counseling). Randomization was performed by the principal investigator at the end of baseline testing using sex-stratified tables. A total of 10 participants were enrolled in each group, with an overall total of 20 participants. For lifestyle counseling, all participants met with registered dietitians who had extensive experience in exercise programming and prescription. They met four times (visits 2–5) for 1 hour each to discuss diet, exercise, and behavior modification based on recommendations in the Diabetes Prevention Program (The Diabetes Prevention Program DPP, 2002). Participants were encouraged to perform at least 150 min per week of moderate-intensity aerobic physical activity and resistance training activities at least twice a week, according to the current American College of Sports Medicine guidelines (ACSM Physical Activity Guidelines, 2003). In addition to the lifestyle counseling intervention described above, those randomized to the semaglutide + lifestyle counseling group received verbal and written instructions on the preparation and self-administration of once weekly subcutaneous semaglutide (Ozempic) injection. The dosage started at 0.25 mg weekly for 4 weeks, then 0.5 mg weekly for 4 weeks, and finally 1.0 mg weekly for the duration of the study (12 weeks). If the titration was not tolerated, the participant remained on the tolerated dose for a longer period and would retry the higher dose at a later visit.

### 2.4 Study outcomes

All outcome measures were collected at baseline and after 20 weeks, with analyses adjusted for baseline values. Assessors were not blinded to group assignments.

#### 2.4.1 Total body weight

Morning fasting weight was measured at each session using a standard, calibrated scale (TANITA WB-3000), without shoes.

#### 2.4.2 Body composition

Total body adipose tissue, lean mass, and BMD of the whole body, leg, pelvis, and lumbar spine were obtained from whole-body dual-energy X-ray absorptiometry (DXA) scans (Horizon, Hologic, Marlborough, MA) at baseline and after 20 weeks. Regional BMD values for the leg, pelvis, and lumbar spine were derived from regions-of-interest analysis of the whole-body scans. All scans were performed in accordance with manufacturer-recommended positioning and analyzed by a trained staff member.

#### 2.4.3 Bone turnover markers

Fasting blood samples were collected at baseline (n = 20) and at the end of the study (n = 20) via venipuncture, following standard procedures, and the serum was stored until analysis. In accordance with the international recommendations, bone formation marker procollagen type I intact N-terminal propeptide (P1NP) and bone resorption marker collagen type I C-telopeptide (CTX) were generated using commercially available chemiluminescence protocols (Immunodiagnostics Systems, The Boldens, United Kingdom), as conducted previously (Wherry et al., 2019).

### 2.5 Covariate assessments

Demographic and medical history (i.e., presence of selected comorbidities) were self-reported at baseline. Analysis of covariance was adjusted for age, sex, and baseline values of each outcome.

### 2.6 Statistical analyses

Baseline characteristics were summarized overall and by a randomized treatment group. Analysis of covariance was adjusted for age, sex, and baseline values to estimate 20-week body weight, BMD, and BTM estimates, along with treatment effects. Partial Pearson correlations were estimated between changes in body weight and changes in whole-body and regional BMD, and adjusted for sex, treatment assignment, age, and baseline values of the outcome BMD. These correlations were then repeated for change in body weight and change in BTMs (CTX and P1NP). Analyses were performed using R version 4.4.2 (R Foundation for Statistical Computing, Vienna, Austria), with *p*-values of 0.05 used to determine statistical significance.

## 3 Results

### 3.1 Participant characteristics

Baseline characteristics were analyzed for 20 participants and are presented as mean ± SD or percentages in Table 1. The participants’ mean age was 72.7 ± 4.8 years, 50% were women, 45% were Hispanic, and the average BMI was 32.9 ± 4.0 kg/m^2^.

**TABLE 1:** Baseline descriptive characteristics of the study sample.

Variable	Overall (n = 20)	Lifestyle counseling alone (n = 10)	Semaglutide + Lifestyle counseling alone (n = 10)
Demographic			
Age (years)	72.7 ± 4.8	73.4 ± 4.9	71.9 ± 5.0
Female, n (%)	10 (50)	5 (50)	5 (50)
White, n (%)	18 (90)	9 (90)	9 (90)
Hispanic, n (%)	9 (45)	4 (40)	5 (50)
Education			
Greater than high school, n (%)	18 (90)	10 (100)	8 (80)
Marital status			
Married, n (%)	12 (60)	7 (70)	5 (50)
Body composition			
Weight (kg)	91.8 ± 16.4	95.7 ± 16.0	87.8 ± 16.7
Body mass index (kg/m^2^)	32.9 ± 4.0	34.1 ± 3.7	31.8 ± 4.1
Presence of comorbidity, n (%)			
Diabetes	6 (30)	5 (50)	1 (10)
Prediabetes	14 (70)	5 (50)	9 (90)
Hypertension	11 (55)	6 (60)	5 (50)
Osteoporosis	4 (20)	2 (20)	2 (20)
DXA-acquired variables			
Total fat mass (kg)	35.41 ± 7.58	37.71 ± 5.88	33.11 ± 8.65
Total lean mass (kg)	51.74 ± 11.44	53.26 ± 11.36	50.23 ± 11.91
Whole body bone mineral density (g/cm^2^)	1.26 ± 0.22	1.33 ± 0.25	1.19 ± 0.16
Lumbar spine bone mineral density (g/cm^2^)	1.18 ± 0.34	1.27 ± 0.41	1.09 ± 0.26
Pelvis bone mineral density (g/cm^2^)	1.26 ± 0.36	1.39 ± 0.43	1.13 ± 0.22
Leg bone mineral density (g/cm^2^)	1.35 ± 0.45	1.51 ± 0.57	1.19 ± 0.22
Biomarkers of bone turnover			
Procollagen type 1 N-terminal propeptide (ug/L)	48.46 ± 26.15	50.01 ± 27.64	46.90 ± 25.96
C-terminal telopeptide of type I collagen (ug/L)	0.28 ± 0.23	0.32 ± 0.28	0.25 ± 0.18

Continuous data are presented as means ± standard deviation, and categorical data are presented as absolute numbers (percentages).

### 3.2 Changes in body weight

**FIGURE 1:**
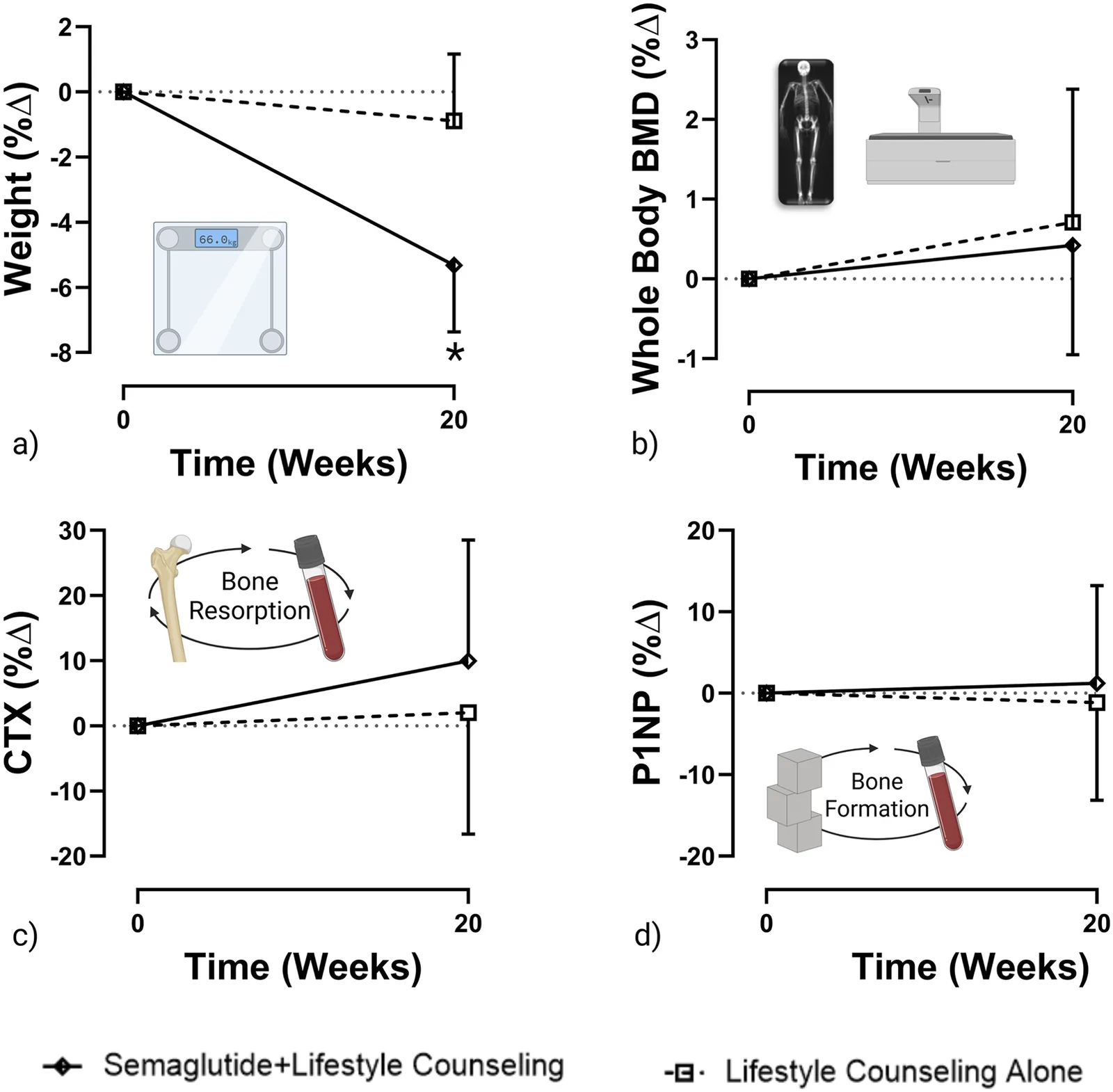
Percent change in weight, whole-body BMD, CTX, and P1NP from baseline to 20 weeks (created with GraphPad Prism and BioRender). Graphs represent **(a)** weight **(b)** whole body BMD **(c)** CTX and **(d)** P1NP percent change from baseline to 20 weeks. *Represents p < 0.05.

Figure 1a presents the percentage change in body weight by group and over time. On average, the semaglutide + lifestyle counseling group lost significantly more weight than the lifestyle counseling alone group [−4.9 kg (95% CI: −6.8 to −3.0) vs. −0.8 kg (95% CI: −2.7 to 1.0); *p* = 0.006], corresponding to −5.32% and −0.89%, respectively.

### 3.3 Changes in bone mineral density

Figure 1b presents the percentage change in whole-body BMD by group and over time. No significant difference in the change in whole-body composition was observed between the semaglutide + lifestyle counseling group [0.005 g/cm^2^ (95% CI: −0.012 to 0.023)] and the lifestyle counseling alone group [0.009 g/cm^2^ (95% CI: −0.009 to 0.03)]; *p* = 0.77, corresponding to 0.42% and 0.71% BMD changes, respectively. Additionally, there were no significant differences in regional BMD changes between the semaglutide + lifestyle counseling group and the lifestyle counseling alone group at the leg, pelvis, or lumbar spine. Mean changes in leg BMD were −0.013 g/cm^2^ (95% CI: −0.038 to 0.012) and −0.005 g/cm^2^ (95% CI: −0.03 to 0.02), respectively (*p* = 0.637), corresponding to −0.98% and −0.36%. Changes in pelvis BMD were −0.039 g/cm^2^ (95% CI: −0.088 to 0.01) and −0.028 g/cm^2^ (95% CI: −0.077 to 0.02), respectively (*p* = 0.75), corresponding to −3.10% and −2.21%. Lumbar spine BMD changes were −0.011 g/cm^2^ (95% CI: −0.157 to 0.136) in the semaglutide group and 0.106 g/cm^2^ (95% CI: −0.041 to 0.25) in the lifestyle-alone group (*p* = 0.26), corresponding to −0.71% and 6.95%, respectively.

### 3.4 Changes in bone turnover markers

Figures 1c,d present percentage changes in CTX and P1NP by group and over time, respectively. No significant changes in BTMs were observed in either group. In the semaglutide + lifestyle counseling group, changes were +0.03 ng/mL for CTX (95% CI: −0.03 to 0.09) and +0.63 ng/mL for P1NP (95% CI: −5.48 to 6.73). In the lifestyle counseling alone group, changes were +0.01 ng/mL for CTX (95% CI: −0.05 to 0.07) and −0.58 mg/mL for P1NP (95% CI: −6.69 to 5.52)]. Between-group comparison showed no significant difference for CTX, *p* = 0.08 (*p-*value based on the log transformation), corresponding to relative changes of 9.96% and 2.02%, or for P1NP, *p* = 0.12 (*p-*value based on the log transformation), corresponding to 1.23% and −1.15%, respectively.

### 3.5 Correlations between weight change and bone change

Partial Pearson correlations were generated to examine whether weight change was associated with changes in BMD or BTMs from baseline to 20 weeks. Weak, nonsignificant correlations were observed for change in weight and change in whole-body BMD (r = 0.25; *p* = 0.35), leg BMD (r = 0.14; *p* = 0.60), and lumbar spine BMD (r = −0.11; *p* = 0.68). A strong, significant direct correlation was discovered for change in weight and change in pelvis BMD (r = 0.62; *p* = 0.01). Weak, inverse correlations were observed for change in weight and change in CTX (r = −0.23; *p* = 0.39) and P1NP (r = −0.26; *p* = 0.34).

## 4 Discussion

In this exploratory *post hoc* analysis of a pilot trial, we aimed to study the effect of GLP1Ra-based therapy on measures of bone health in older adults with overweight or obesity. We observed that participants in the semaglutide + lifestyle counseling group lost significantly more weight than those in the lifestyle counseling alone group (−5.3% compared to −0.9%); however, no differences were observed for BMD or BTMs by group or over time. Although this was an exploratory *post hoc* analysis, it is noteworthy that observed group differences suggested a trend toward bone loss, with consistently lower BMD and increased bone turnover at follow-up in the semaglutide + lifestyle counseling group compared to that in the lifestyle counseling alone. Finally, a significant, direct correlation was observed between weight change and change in BMD at the pelvis.

So far, preclinical and observational data examining the effects of GLP1Ra-based therapies on bone suggest a positive association, whereby GLP1Ra use reduces turnover and promotes bone formation (Daniilopoulou et al., 2022; Tan et al., 2025) and may reduce the fracture risk in some populations (Xie et al., 2025; Zhang et al., 2025). Clinical trial evidence reporting on BMD and BTMs is currently emerging. Our findings herein align with a small meta-analysis published by Kim et al. in 2024, which synthesized data across seven randomized controlled trials (RCTs) reporting on BMD and BTMs in participants (average age: 51.7 years) allocated to either GLP1Ra or placebo/control for an average of 33.7 weeks. No effects of GLP1Ra-based treatment on regional BMD were observed. The mean difference (MD) at each site was as follows: femoral neck, +0.01 g/cm^2^ (95% CI: −0.01 to 0.04); total hip, −0.01 g/cm^2^ (95% CI: −0.02 to 0.01); and lumbar spine, 0 g/cm^2^ (95% CI: −0.02 to 0.02); however, there was a small increase in CTX [MD: +0.04 μg/L (95% CI: 0.01 to 0.07)] (Kim et al., 2024).

Weight loss attributable to the GLP1Ra interventions included in the meta-analysis by Kim et al. was modest, with most values hovering approximately 2%–4% (Kim et al., 2024). Given this, and consistent with data indicating that weight loss of 10% has been shown to yield 1%–2% bone loss (Kim et al., 2024), the expected bone loss attributable to 5% weight loss would be expected to range from 0.5% to 1%, a change that falls within the typical margin of error of a DXA machine (Shapses and Riedt, 2006) and less than what would be considered clinically meaningful [e.g., 2%–3% per year (Lewiecki, 2024)]. Only one trial (Hansen et al., 2024) reported more substantial weight loss (i.e., >8%) in the semaglutide group, with an estimated treatment difference (ETD) of 8.8% weight loss over 52 weeks. Additionally, a lower total hip BMD [ETD: −0.020 g/cm^2^ (95% CI: −0.032 to −0.008); p = 0.001] and lumbar spine BMD [ETD: −0.018 g/cm^3^ (95% CI: −0.031 to −0.005); p = 0.007], along with higher CTX [ETD: 166.4 ng/L (95% CI 25.5–307.3); p = 0.021] in the semaglutide group versus placebo group were observed (Hansen et al., 2024). Similarly, the Semaglutide and Cardiovascular Outcomes in Obesity without Diabetes Trial randomized 17,604 individuals to 2.4 mg/week semaglutide or a placebo. This trial, which included 40+ months of follow-up, reported an ETD in weight of −8.5% at 104 weeks (Lincoff et al., 2023) and found nearly a 5-fold higher incidence of hip and pelvic fractures in adults aged 75 years or older using semaglutide compared with placebo (Wegovy FDA, 2019). This suggests that GLP1Ra treatment which induced at least an 8% weight loss showed significant bone loss, and the null findings in this exploratory *post hoc* analysis may, in part, reflect the modest weight loss achieved over a relatively short intervention period, particularly when compared with outcomes observed in larger, longer-term trials. Indeed, our correlation analysis which suggests that greater weight loss is associated with greater pelvis BMD loss do align with prior work (Zibellini et al., 2015) and support future research to evaluate the effect of GLP1Ra use on skeletal health outcomes, particularly with GLP1Ra-based therapies capable of producing greater weight loss in older adults.

Another important consideration when evaluating the impact of these medications on bone health outcomes is the sustainability of GLP1Ra use as weight regain following cessation of treatment is expected (Quarenghi et al., 2025). Analogous data from lifestyle-based and surgical weight loss suggest that weight regain is not associated with bone regain (Waters et al., 2013; Gagnon and Schafer, 2018). External factors such as cost and adverse events can influence long-term use of GLP1Ra-based treatments. Subcutaneous GLP1Ra-based obesity treatment is expensive, net prices ranging from $717 to $761 per month, with further variations based on insurance coverage (Hernandez and Sullivan, 2024). Currently, Medicare covers GLP1Ra medications that are approved for type 2 diabetes and cardiovascular disease through Part D plans, but not for obesity treatment (Medicare coverage, 2024). The likelihood of discontinuing treatment is high, especially in patients aged 65 years and older with type 2 diabetes: hazard ratio [HR], 1.28 [95% CI: 1.24–1.32]; without type 2 diabetes: HR, 1.18 [95% CI: 1.13–1.22] (Rodriguez et al., 2025). This highlights the need for further research on the evaluation of the impact of intermittent GLP1Ra use on bone health outcomes.

To our knowledge, this is the first randomized trial to report clinically relevant bone outcomes following GLP1Ra use in older adults. Strengths include the utilization of clinically relevant bioimaging and biomarker data to assess change in bone health, along with the inclusion of men and persons identified as Hispanic, as both tend to be under-represented in weight loss trials (Beavers et al., 2020). Several limitations are worth noting, including the lack of assessor and participant blinding, small sample size, and the short duration, all of which need to be considered when interpreting BMD outcomes and in the planning of future work. Furthermore, dietary adherence and physical activity levels were not monitored throughout the trial. This limits the ability to account for important variables, such as specific nutrient intake (e.g., calcium and protein) and changes in exercise patterns, which may independently influence skeletal health outcomes. Given the pilot nature of the trial, the (relatively) small degree of weight loss achieved, and the well-described association between weight loss and fracture risk, we believe that further exploration into this increasingly relevant question is warranted.

## 5 Conclusion

In conclusion, results from this pilot trial demonstrate that 20 weeks of semaglutide combined with lifestyle counseling led to approximately 5% greater weight loss than that achieved with lifestyle counseling alone. However, no significant changes in BMD or BTMs were observed. Research on the effects of GLP1Ra-based therapies on the skeletal system are still emerging; therefore, adopting strategies to minimize muscle and bone loss is prudent. Known countermeasures to weight loss-associated bone loss include dietary factors, ensuring adequate protein, calcium, and vitamin D, along with participating in progressive resistance training (RT) to support skeletal muscle health and maintain long-term weight loss (Beavers et al., 2024). Given the rapid, widespread adoption of GLP1Ra-based therapies and their increasing potential to induce clinically significant weight loss in older adults, large-scale, long-term trials are needed to define the true risk–benefit profile for skeletal health and inform strategies to mitigate fracture risk.

## Data Availability

The raw data supporting the conclusions of this article will be made available by the authors, without undue reservation.

## Abbreviations

BMI, body mass index; BMD, bone mineral density; GLP1Ra, glucagon-like peptide-1 receptor agonist; FDA, Food and Drug Administration; DXA, dual-energy X-ray absorptiometry; BTMs, bone turnover markers; P1NP, procollagen type I intact N-terminal propeptide; CTX, collagen type I C-telopeptide; ETD, estimated treatment difference; RCT, randomized controlled trial; RT, resistance training.
