# Correction: Bone mineral density and turnover response to GLP-1 receptor agonists in older adults with overweight/obesity and prediabetes/type 2 diabetes: a 20-week pilot trial *post hoc* analysis

**DOI:** 10.3389/fragi.2026.2016735

**Published:** 2026-10-19

**Authors:** Lauren Dinkla, Kristen M. Beavers, Ronna Robbins, Dela Akpalu, Sarah J. Wherry, Gary Miller, Daniel P. Beavers, Sara Espinoza, Jonathan Trejo, Allison Stepanenko, Tiffany M. Cortes

**Affiliations:** 1 Department of Health and Exercise Science, Wake Forest University, Winston-Salem, NC, United States; 2 Department of Nutrition and Food Sciences, Texas Woman’s University, Denton, TX, United States; 3 Department of Statistical Science, Wake Forest University, Winston-Salem, NC, United States; 4 Division of Geriatric Medicine, University of Colorado Anschutz Medical Campus, Aurora, CO, United States; 5 VA Eastern Colorado Geriatric Research, Education, and Clinical Center (GRECC), VA Eastern Colorado Healthcare System, Aurora, CO, United States; 6 Center for Translational Geroscience, Department of Medicine, Cedars-Sinai Medical Center, Los Angeles, CA, United States; 7 Diabetes and Aging Center, Department of Medicine, Cedars-Sinai Medical Center, Los Angeles, CA, United States; 8 Department of Medicine, UT Health San Antonio, Sam and Ann Barshop Institute for Longevity and Aging Studies, UT Health San Antonio, San Antonio, TX, United States; 9 Endocrinology Section, South Texas Veterans Healthcare System, San Antonio, TX, United States; 10 San Antonio Geriatric Research, Education, and Clinical Center (GRECC), South Texas Veterans Healthcare System, San Antonio, TX, United States

**Keywords:** glucagon‐like peptide‐1 receptor agonist, randomized controlled trial, older adults, bone mineral density, bone turnover

There was a mistake in Table 1 as published. The column headers for the study arms were transposed: the baseline data displayed under Semaglutide + Lifestyle Counseling belong to Lifestyle Counseling Alone, and *vice versa*. The corrected Table 1 appears below.

**TABLE 1:** Baseline descriptive characteristics of the study sample.

Variable	Overall (n = 20)	Lifestyle counseling alone (n = 10)	Semaglutide + Lifestyle counseling alone (n = 10)
Demographic			
Age (years)	72.7 ± 4.8	73.4 ± 4.9	71.9 ± 5.0
Female, n (%)	10 (50)	5 (50)	5 (50)
White, n (%)	18 (90)	9 (90)	9 (90)
Hispanic, n (%)	9 (45)	4 (40)	5 (50)
Education			
Greater than high school, n (%)	18 (90)	10 (100)	8 (80)
Marital status			
Married, n (%)	12 (60)	7 (70)	5 (50)
Body composition			
Weight (kg)	91.8 ± 16.4	95.7 ± 16.0	87.8 ± 16.7
Body mass index (kg/m^2^)	32.9 ± 4.0	34.1 ± 3.7	31.8 ± 4.1
Presence of comorbidity, n (%)			
Diabetes	6 (30)	5 (50)	1 (10)
Prediabetes	14 (70)	5 (50)	9 (90)
Hypertension	11 (55)	6 (60)	5 (50)
Osteoporosis	4 (20)	2 (20)	2 (20)
DXA-acquired variables			
Total fat mass (kg)	35.41 ± 7.58	37.71 ± 5.88	33.11 ± 8.65
Total lean mass (kg)	51.74 ± 11.44	53.26 ± 11.36	50.23 ± 11.91
Whole body bone mineral density (g/cm^2^)	1.26 ± 0.22	1.33 ± 0.25	1.19 ± 0.16
Lumbar spine bone mineral density (g/cm^2^)	1.18 ± 0.34	1.27 ± 0.41	1.09 ± 0.26
Pelvis bone mineral density (g/cm^2^)	1.26 ± 0.36	1.39 ± 0.43	1.13 ± 0.22
Leg bone mineral density (g/cm^2^)	1.35 ± 0.45	1.51 ± 0.57	1.19 ± 0.22
Biomarkers of bone turnover			
Procollagen type 1 N-terminal propeptide (ug/L)	48.46 ± 26.15	50.01 ± 27.64	46.90 ± 25.96
C-terminal telopeptide of type I collagen (ug/L)	0.28 ± 0.23	0.32 ± 0.28	0.25 ± 0.18

Continuous data are presented as means ± standard deviation, and categorical data are presented as absolute numbers (percentages).

The original version of this article has been updated.

